# Neurofilament Heavy Chain and Tau Protein Are Not Elevated in Cerebrospinal Fluid of Adult Patients with Spinal Muscular Atrophy during Loading with Nusinersen

**DOI:** 10.3390/ijms20215397

**Published:** 2019-10-30

**Authors:** Andreas Totzeck, Benjamin Stolte, Kathrin Kizina, Saskia Bolz, Melina Schlag, Andreas Thimm, Christoph Kleinschnitz, Tim Hagenacker

**Affiliations:** Department of Neurology, University Hospital Essen, Hufelandstr. 55, 45147 Essen, Germany; benjamin.stolte@uk-essen.de (B.S.); kathrin.kizina@uk-essen.de (K.K.); saskia.bolz@uk-essen.de (S.B.); melina.schlag@uk-essen.de (M.S.); christoph.kleinschnitz@uk-essen.de (C.K.)

**Keywords:** SMA, antisense oligonucleotide, amyotrophic lateral sclerosis, motor neuron disease

## Abstract

Nusinersen is the first approved drug for the treatment of spinal muscular atrophy (SMA). Treatment of SMA with nusinersen is based on a fixed dosing regimen. For other motoneuron diseases, such as amyotrophic lateral sclerosis (ALS), biomarkers are available for clinical diagnostics; however, no such biomarkers have yet been found for SMA. Serum and cerebrospinal fluid (CSF) samples of 11 patients with adult SMA type 3 were prospectively collected and analyzed during loading with nusinersen. Neurofilament heavy chain, tau protein, S100B protein, and neuron-specific enolase were investigated as potential biomarkers of motor neuron destruction. No significant pathological alterations in levels of neurofilament heavy chain, tau protein, or S100B protein were detected in the CSF or blood samples under baseline conditions or during loading with nusinersen. Neuron-specific enolase was marginally elevated in CSF and blood samples without significant alteration during treatment. In a mixed cohort of adult patients with SMA type 3, neurofilament heavy chain, tau protein, S100B protein, and neuron-specific enolase do not serve as potential biomarkers during the loading phase of nusinersen. The slow progression rate of SMA type 3 may not lead to detectable elevation of levels of these common markers of axonal degradation.

## 1. Introduction

Spinal muscular atrophy (SMA) is an autosomal recessive neuromuscular disorder characterized by atrophy and weakness of skeletal muscles caused by mutations of the survival motor neuron 1 gene (*SMN1*) [1,2,3]. The survival motor neuron (SMN) protein is ubiquitously expressed but serves a predominant function in spinal motor neurons. Homozygous deletions or loss-of-function mutations at locus 5q13 result in insufficient expression of the SMN protein [2,4]. A paralogous gene, *SMN2*, also encodes for a truncated SMN protein that is dysfunctional due to a splice site variant in *SMN2*, leading to exclusion of exon 7 from the mature RNA [5].

The classification of SMA is based on age at symptom onset and the most advanced milestone attained during motor development. Patients with SMA type 3 reveal symptoms after 18 months of age and gain the ability to walk independently at some point [6,7].

The first approved drug for the treatment of SMA, nusinersen, is an antisense oligonucleotide, which is intrathecally administered. Nusinersen binds to a specific sequence within the *SMN2* premessenger RNA and modifies its splicing to promote the expression of full-length functional SMN protein. In clinical trials, treatment with nusinersen significantly increased motor function and survival in infants and children with SMA type 1 or 2 [8,9]. Based on these results, nusinersen was approved by the US Food and Drug Administration (FDA) in December 2016 and by the European Medicines Agency (EMA) in May 2017 for treatment of SMA in infants and adults.

Treatment with nusinersen involves a fixed dosing regimen based on trials in infants and children with SMA type 1 or 2 and does not consider the clinical course of SMA, the progression rate of disease, or laboratory findings. In infants with SMA type 1, phosphorylated neurofilament heavy chain (NF-H) and neurofilament light chain were elevated before treatment and decreased during treatment with nusinersen [10,11]. To date, there is little data regarding treatment of adult patients. In amyotrophic lateral sclerosis (ALS), elevated levels of NF-H reflect axonal destruction and have recently been established as a biomarker that can help confirm the diagnosis. In SMA, similar biomarkers have yet to be identified in adults and could help determine disease progression rate and response to medical treatment.

We prospectively collected and analyzed serum and cerebrospinal fluid (CSF) samples from the first 11 adult patients with SMA type 3 treated with nusinersen in our cohort in Germany. Similar to the biomarker studies of ALS, NF [12,13,14,15,16,17], tau protein, S100B protein (S100B), and neuron-specific enolase (NSE) were investigated as potential biomarkers of motor neuron destruction [18,19,20,21,22].

## 2. Results

### 2.1. Clinical Characterization

**Figure 1:**
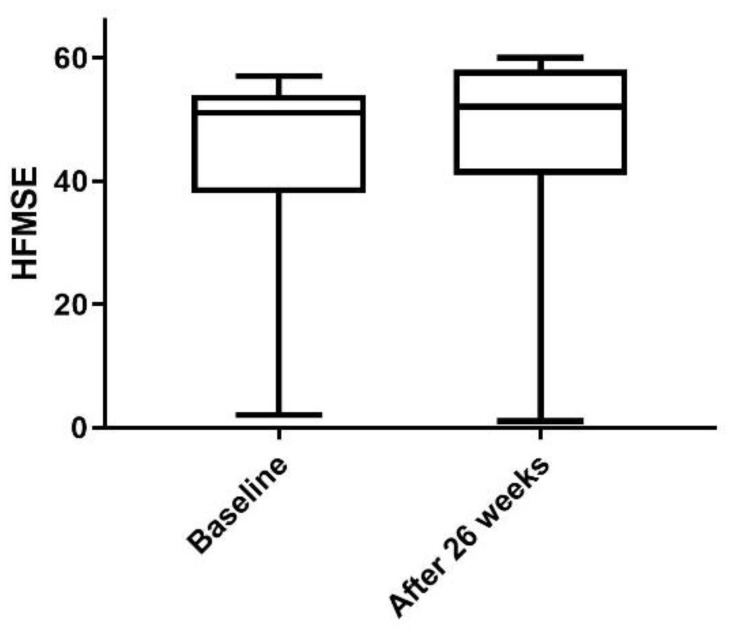
Changes in HFMSE in all 11 patients measured before the first administration and four months after the fourth administration of nusinersen. The box plot shows minimum to maximum range and mean.

Physical and neurological examination, lumbar puncture, and nusinersen administration were carried out at the Department of Neurology of the University Hospital in Essen, Germany. All 11 patients showed stability or a trend toward improvement on the Hammersmith Functional Motor Scale Expanded (HFMSE) for SMA, which was measured before the first and the fifth administration of nusinersen (Table 1 and Figure 1).

### 2.2. Laboratory Results

In all CSF samples but one (Patient 11), red blood cell count (RBC) was below 500/µL. Eight CSF samples showed RBC below 100/µL (Table 2). RBC was elevated in one blood sample of Patient 5 (5.9/pL), and RBC in all other blood samples was within the reference range (4.5–5.6/pL). White blood cell count (WBC) was elevated in one blood sample of Patient 5 (11.87/nL) and one blood sample of Patient 9, who initially presented with elevated WBC in CSF. WBC was within the reference range in all other blood samples (3.6–9.2/nL). Lactate levels in seven out of 44 samples (measured in sodium fluoride plasma) were slightly elevated up to 3.3 mM, while all others were within the reference range (0.5–2.2 mM). CSF lactate was within the reference range in all 44 samples (1.2–2.1 mM). Glucose levels (measured in sodium fluoride/citrate plasma) were marginally elevated in nine of the 44 samples from five different patients (101–131 mg/dL, reference range 74–99 mg/dL). In all CSF samples, glucose was within the reference range (49–75 mg/dL). In all four of the CSF samples from the five older male patients (Patients 1, 3, 5, 9, and 11), the total protein was elevated. The other patients showed CSF protein levels within the reference range.

**Figure 2:**
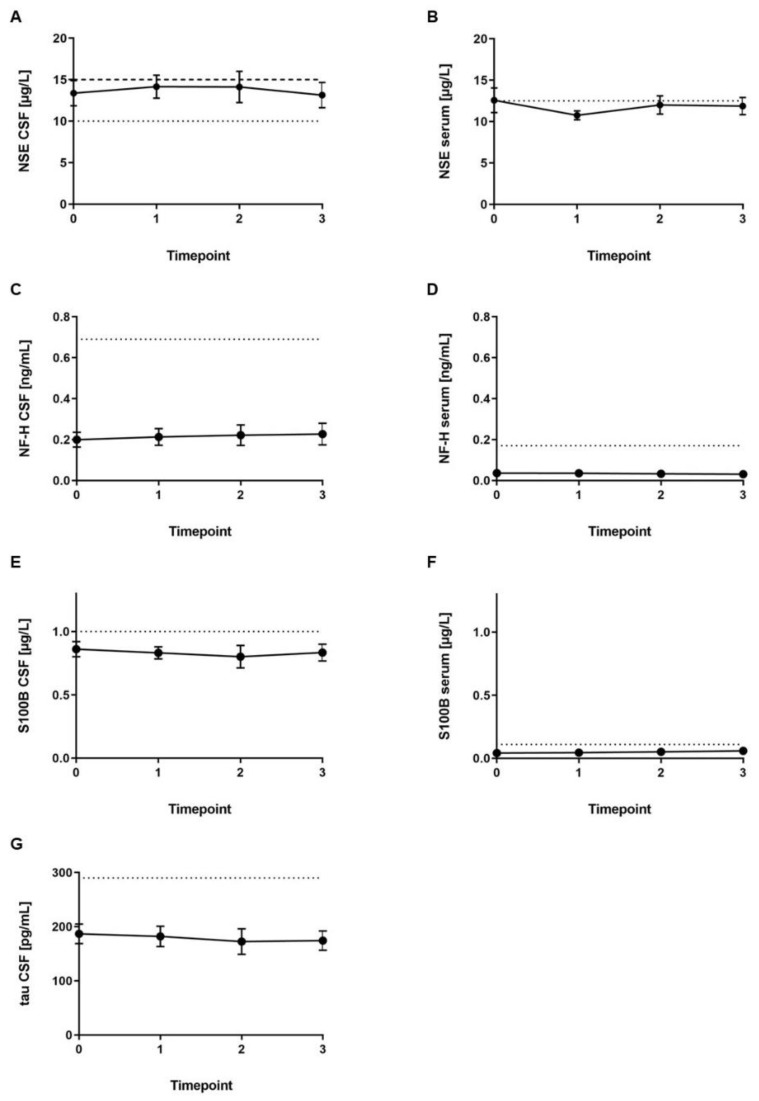
Mean levels (± SEM) of biomarkers measured at four timepoints during loading with nusinersen. (**A**) Neuron-specific enolase (NSE) in cerebrospinal fluid (CSF), (**B**) NSE in serum, (**C**) neurofilament heavy chain (NF-H) in CSF, (**D**) NF-H in serum, (**E**) S100B protein (S100B) in CSF, (**F**) S100B in serum, (**G**) tau protein in CSF. Timepoint 0 = before first administration, timepoint 1 = 14 days after first administration and before second, timepoint 2 = 14 days after second administration and before third, timepoint 3 = 30 days after third administration and before fourth. Reference range is indicated by dotted lines. Reference range for NSE in the CSF for ≤45 years of age (<10 µg/L) is indicated by dotted lines, and reference range for >45 years of age (<15 µg/L) is indicated by dashed lines.

CSF tau protein (reference range: <290 pg/mL), CSF NF-H (reference range: <0.69 ng/mL), and serum NF-H (reference range: <0.17 ng/mL) levels were within the reference range in all patients (Figure 2). Serum S100B was marginally increased in one sample (Patient 5; reference range: <0.11 µg/L). S100B level in CSF was slightly increased in three out of four CSF samples from one patient (Patient 3; reference range: <1.0 µg/L). Serum NSE levels were increased in three out of four samples of Patient 5 and slightly increased in four samples out of three patients (reference range: <12.5 µg/L). NSE level in the CSF was marginally increased in 18 out of 20 samples from the four male patients and female Patient 6 (reference range for age ≤45 years: <10 µg/L; reference range for age >45 years: 15 µg/L).

Analysis of CSF to serum ratio of NSE, NF-H, and S100B revealed no further significant information (data not shown). There was no correlation between CSF NSE level and clinical presentation.

## 3. Discussion

This is the first pilot study examining blood and CSF samples as possible biomarkers during loading with nusinersen in adult patients with SMA type 3.

The stability or trend toward improvement on the HFMSE measured before the first and the fifth doses of nusinersen may indicate a positive response to the nusinersen treatment. However, a larger group of patients and a longer time of treatment are needed to clarify treatment response to nusinersen in adult patients with SMA type 3.

While the five older patients showed elevated CSF total protein levels, the younger patients had CSF total protein levels within the reference range. Elevated CSF total protein can be found in patients with neuroinflammatory or neurodegenerative disorders like multiple sclerosis, chronic inflammatory demyelinating polyneuropathy (CIDP), or Alzheimer’s disease [19,23,24,25]. In SMA type 3, there are no reports regarding the CSF total protein levels during the course of the disease in adult patients. However, we found that this parameter was not specific and did not vary during dosing with nusinersen. WBC in CSF was marginally elevated in seven patients as possible reactive pleocytosis due to repeated lumbar puncture for nusinersen administration.

In our patients, tau protein and NF-H levels were not elevated in the CSF. Both proteins are well-established markers of possible neural destruction. Elevated levels of NF in the CSF facilitate diagnosis of ALS [12,13,14,17]. Tau protein in the CSF marks neurodegeneration and progression, especially in Alzheimer’s disease [19]. In a recent study, total CSF tau protein correlated with the progression of ALS, suggesting shorter survival time with high levels of tau protein [21]. Here, we measured the levels of NF-H and total tau protein but not those of NF light chain or phospho-tau protein. NF light chain is considered an indicator of neurodegeneration in ALS, similar to heavy chain [21,26,27], while NF-H is a more well-established parameter. Recent studies revealed no increase in NF-H and NF light chain after at least four months of treatment with nusinersen in adults with SMA type 2 and 3 [28,29]. However, data of NF during loading with nusinersen in adults with SMA type 3 is still lacking. This study’s results support the negligible role of NF-H in adult patients with SMA type 3. In addition, all tau protein levels in our cohort were within the reference range. Tau protein is a marker of destruction in dementia and cerebrovascular disease [30]. An increase in phospho-tau protein level along with elevated tau protein level helps to differentiate between dementias, while in acute stroke, tau protein (but not phospho-tau protein) level is elevated [31]. Therefore, considering our findings of tau protein levels within the reference range present in CSF, we did not assay for the presence of phospho-tau. A recent study suggested the biomarker potential of phospho-tau in acute and chronic traumatic brain injury; further data is needed to confirm recent findings and determine its usefulness in this context [32]. In infants with SMA type 1, elevated levels of NF-H and light chain were found to decrease during treatment with nusinersen [10,11]. Young patients with SMA type 1 are more severely and rapidly affected than adults with SMA type 3. This pilot study did not find elevated levels of NF-H; thus, the role of axonal destruction in adult patients with SMA type 3 is not as predominant as in SMA type 1. However, due to the small sample size, further studies with a higher number of patients and performed in different centers should be considered to establish the role of this biomarker in SMA.

S100B level in the CSF was slightly elevated in Patient 3, and NSE levels were at some point marginally elevated in all patients except the youngest (Patient 4) and the oldest (Patient 11) patient. S100B and NSE are used as biomarkers for neuronal damage and are directly related to disease progression in patients with acute brain injury [20,33]. Furthermore, S100B—mainly concentrated in astrocytes—is recognized as a reliable biomarker of active neuronal distress in Alzheimer’s disease, Parkinson’s disease, ALS, or multiple sclerosis [18]. In the peripheral nervous system, S100B is indicative of axonal degeneration and regeneration [22] and of inflammatory autoimmune disorders, such as CIDP [25]. In our patients, marginally elevated S100B and NSE levels may reflect axonal damage; however, findings were unspecific, and levels did not alter during the loading with nusinersen. However, assessing a possible decline of NSE after a longer period of treatment with nusinersen was not the goal of this study, and it will thus need further examination. The moderately increased levels of S100B and NSE as well as reference levels of NF and tau protein possibly result from the slow and progressive nature of SMA type 3. Neuronal destruction markers seen in highly active and rapidly destructive diseases like ALS may not be suitable markers for SMA type 3, or if they are, different reference levels must be established. The assays employed may also not be sensitive enough to detect small changes, although they are the most sensitive assays available at this time. Furthermore, longer observation periods may be required to detect changes during ongoing treatment with nusinersen. Recent studies have revealed that SMA is a multiorgan disorder, which leads to impairment of lymphoid organ function. This may have an impact on neuroinflammatory processes and contribute to the pathogenesis of SMA [34,35]. As such, a novel strategy may be to identify biomarkers for neuroinflammation, rather than neuronal damage, in patients with SMA type 3. Furthermore, if adult patients with SMA type 3 clinically stabilize or improve during nusinersen treatment, biomarkers for reactivation of “hibernating” motor neurons or synaptogenesis may correlate with regaining of motor function and could prove more relevant than destruction markers. Possible candidates include thrombospondins, which are major synaptogenic factors secreted by astrocytes [36,37]. Advanced methods like unbiased proteomics of CSF may help identify other soluble biomarkers in the future.

## 4. Materials and Methods

### 4.1. Patients

In this pilot study, 11 patients with SMA type 3 were neurologically examined for neuromuscular diseases in our clinic at the Department of Neurology of the University Hospital in Essen, Germany, between 2017 and 2019. The group consisted of four female and seven male patients. SMA type 3 was diagnosed in all patients, and genetic testing was done prior to their first visit. There was a documented mutation of *SMN1* (i.e., 5q-SMA) and copy number of *SMN2* in all patients (Table 1). Nusinersen treatment was initiated with four loading doses: the first three loading doses were administered at 14-day intervals, and the fourth loading dose was administered 30 days after the third dose. The fifth dose was given four months later [38]. The recommended dosage of 12 mg (5 mL) nusinersen was administered. Each patient was examined by a neurologist prior to each administration. The HFMSE was used to measure motor function before the first application and the fifth dose of nusinersen. Informed consent was obtained from every patient, following which blood and CSF samples were collected before each dose. This study was approved by the Ethics Committee of the University Duisburg-Essen, Germany (approval number: 18-8071-BO).

### 4.2. Laboratory Analysis

Blood and CSF samples were taken before each dose of nusinersen. Each sample was either directly examined for blood and CSF cell counts as well as CSF lactate, glucose, and total protein levels or immediately stored at −80 °C for further shipment. Freezing and thawing were not considered to alter the results of the applied assays.

Blood and CSF cell counts as well as CSF lactate, glucose, and total protein levels were analyzed in the laboratory of the University Hospital Essen, Germany.

NSE and S100B levels in frozen CSF and serum and tau protein levels in frozen CSF were analyzed in the laboratory of Dr. Limbach and colleagues, Heidelberg, Germany.

The NSE assay was based on time-resolved amplified cryptate emission (TRACE) technology on a KRYPTOR analyzer (BRAHMS, Hennigsdorf, Germany) [39,40]. S100B protein levels were measured with an electrochemiluminescence immunoassay for the quantification of protein (ECLIA kit, Roche Diagnostics, Mannheim, Germany) [41,42]. For measurement of tau protein level, a standard enzyme-linked immunosorbent assay (ELISA) was performed (Euroimmun AG, Lübeck, Germany) [43].

The Clinical Immunological Laboratory Dr. Stöcker, Lübeck, Germany, analyzed NF levels in frozen CSF and serum samples. Phosphorylated NF-H level was measured using an established ELISA (Euroimmun AG, Lübeck, Germany) [44].

Reference range was defined and established by each of the German reference laboratories compared to the normal population.

### 4.3. Statistics

Mean values and SEM of laboratory results were calculated with PRISM 8 (GraphPad Software, San Diego, CA, USA).

## 5. Conclusions

In this cohort of adult patients with SMA type 3, neurofilament heavy chain, tau protein, S100B protein, and neuron-specific enolase did not seem to serve as potential biomarkers in the early treatment phase during loading with nusinersen. However, the varying clinical presentation of our patients may mask subgroup changes that will need examination of larger cohorts to clarify. Furthermore, the relatively slow progression rate of SMA type 3 compared to that of ALS may not lead to detectable elevation of levels of these common markers of axonal degradation. We propose that unbiased proteome studies and identification of neuroinflammatory markers or markers of synaptogenesis may prove more useful.

## Figures and Tables

**Table 1:** Clinical data on Patients 1–11, including test values of Hammersmith Functional Motor Scale Expanded (HFMSE).

	Patient 1	Patient 2	Patient 3	Patient 4	Patient 5	Patient 6	Patient 7	Patient 8	Patient 9	Patient 10	Patient 11
Age [y]	39	29	48	19	38	28	44	29	51	27	71
Gender	male	male	male	female	male	female	female	male	male	female	male
*SMN2* gene copy number [n]	4	4	3	3	4	4	4	4	7	3	4
Age when diagnosed [y]	28	15	36	9	15	11	2	13	16	19	4
Working	yes	yes	yes	yes	yes	yes	yes	yes	yes	yes	no
Able to walk	yes	yes	no	yes	yes	yes	yes	yes	yes	yes	no
HFMSE [n of 66] timepoint 0	51	54	16	53	44	57	51	57	38	38	2
HFMSE [n of 66] before fifth dose	53	59	36	60	45	59	52	57	41	45	1

**Table 2:** White blood cell count (WBC), red blood cell count (RBC), and levels of lactate, glucose, and total protein in Patients 1–11 measured at four timepoints during loading with nusinersen (timepoint 0 = before first administration, timepoint 1 = 14 days after first administration and before second, timepoint 2 = 14 days after second administration and before third, timepoint 3 = 30 days after third administration and before fourth). Levels out of reference range are highlighted in bold.

	Timepoint	Patient 1	Patient 2	Patient 3	Patient 4	Patient 5	Patient 6	Patient 7	Patient 8	Patient 9	Patient 10	Patient 11
CSF WBC (/µL)	0	**5**	2	3	1	**5**	3	3	4	**6**	1	1
Reference range: <5/µL	1	0	0	3	3	**9**	**5**	3	3	4	2	**5**
	2	1	3	**6**	**5**	**6**	3	3	**6**	**6**	2	1
	3	1	3	3	2	4	3	1	4	3	3	0
CSF RBC (/µL)	0	<100	<100	<100	<100	<500	<500	<500	<500	<500	<500	<500
	1	<100	<100	<100	200	<500	<500	<500	<500	<500	<500	<500
	2	<100	100	<100	<500	<500	<500	<500	<500	<500	<500	**700**
	3	100	<500	100	<500	<500	<500	<500	<500	<500	<500	<500
CSF lactate (mM)	0	1.6	1.7	1.6	1.7	1.7	1.4	1.7	1.7	1.8	1.7	2.5
Reference range: 0.5–2.2 mM,	1	1.3	1.3	1.7	1.5	1.8	1.6	1.6	1.5	1.6	1.8	2.3
>60 years: 1.7–2.6 mM	2	1.4	1.4	1.4	1.5	1.6	1.7	1.9	1.5	1.9	1.7	2.1
	3	1.4	1.5	1.8	1.5	1.6	1.7	1.7	1.3	2.0	1.9	2.1
CSF glucose (mg/dL)	0	64	70	64	59	63	60	60	66	69	60	60
Reference range: 49–75 mg/dL	1	61	63	65	58	59	61	56	66	66	59	61
	2	58	67	60	57	63	58	60	61	70	61	55
	3	63	68	70	59	65	62	58	61	63	61	60
CSF total protein (mg/dL)	0	**63**	34	**63**	27	**56**	36	40	29	**54**	36	**68**
Reference range: 15–45 mg/dL	1	**60**	24	**62**	33	**56**	35	43	31	**55**	32	**70**
	2	**66**	36	**63**	31	**50**	36	40	29	**55**	25	**80**
	3	**69**	34	**58**	27	**50**	30	41	31	**57**	31	**57**

## Abbreviations

ALS	amyotrophic lateral sclerosis
CSF	cerebrospinal fluid
HFMSE	Hammersmith Functional Motor Scale Expanded
NF	neurofilament
RBC	red blood cell count
SEM	standard error of mean
SMA	spinal muscular atrophy
SMN	survival motor neuron
WBC	white blood cell count
